# Overcoming the challenges of drug development in platinum-resistant ovarian cancer

**DOI:** 10.3389/fonc.2023.1258228

**Published:** 2023-10-17

**Authors:** Ramez N. Eskander, Kathleen N. Moore, Bradley J. Monk, Thomas J. Herzog, Christina M. Annunziata, David M. O’Malley, Robert L. Coleman

**Affiliations:** ^1^Division of Gynecologic Oncology, Department of Obstetrics and Gynecology, Rebecca and John Moores Cancer Center, University of California San Diego Health, San Diego, CA, United States; ^2^Gynecologic Oncology, Stephenson Cancer Center, The University of Oklahoma College of Medicine, Oklahoma, OK, United States; ^3^Gynecologic Oncology, HonorHealth Research Institute, University of Arizona College of Medicine, Creighton University School of Medicine, Phoenix, AZ, United States; ^4^Obstetrics and Gynecology, University of Cincinnati Cancer Center, Cincinnati, OH, United States; ^5^Center for Cancer Research, National Cancer Institute, Bethesda, MD, United States; ^6^Division of Gynecologic Oncology, The Ohio State University and The James Comprehensive Cancer Center, Columbus, OH, United States; ^7^Gynecologic Oncology, US Oncology Research, Texas Oncology, The Woodlands, TX, United States

**Keywords:** bevacizumab, biomarker, folate receptor alpha, mirvetuximab soravtansine, platinum-resistant ovarian cancer, and targeted therapy

## Abstract

The definition of “platinum-resistant ovarian cancer” has evolved; it now also reflects cancers for which platinum treatment is no longer an option. Standard of care for platinum-resistant ovarian cancer is single-agent, non-platinum chemotherapy with or without bevacizumab, which produces modest response rates, with the greatest benefits achieved using weekly paclitaxel. Several recent phase 3 trials of pretreated patients with prior bevacizumab exposure failed to meet their primary efficacy endpoints, highlighting the challenge in improving clinical outcomes among these patients. Combination treatment with antiangiogenics has improved outcomes, whereas combination strategies with immune checkpoint inhibitors have yielded modest results. Despite extensive translational research, there has been a lack of reliable and established biomarkers that predict treatment response in platinum-resistant ovarian cancer. Additionally, in the platinum-resistant setting, implications for the time between the penultimate dose of platinum therapy and platinum retreatment remain an area of debate. Addressing the unmet need for an effective treatment in the platinum-resistant setting requires thoughtful clinical trial design based on a growing understanding of the disease. Recent cancer drug approvals highlight the value of incorporating molecular phenotypes to better define patients who are more likely to respond to novel therapies. Clinical trials designed per the Gynecologic Cancer InterGroup recommendations—which advocate against relying solely upon the platinum-free interval—will help advance our understanding of recurrent ovarian cancer response where platinum rechallenge in the platinum-resistant setting may be considered. The inclusion of biomarkers in clinical trials will improve patient stratification and potentially demonstrate correlations with biomarker expression and duration of response. With the efficacy of antibody-drug conjugates shown for the treatment of some solid and hematologic cancers, current trials are evaluating the use of various novel conjugates in the setting of platinum-resistant ovarian cancer. Emerging novel treatments coupled with combination trials and biomarker explorations offer encouraging results for potential strategies to improve response rates and prolong progression-free survival in this population with high unmet need. This review outlines existing data from contemporary clinical trials of patients with platinum-resistant ovarian cancer and suggests historical synthetic benchmarks for non-randomized trials.

## 1 Introduction: Current landscape in platinum-resistant ovarian cancer

Although surgery and platinum-based chemotherapy are effective treatment strategies for advanced-stage epithelial ovarian cancer, most tumors will relapse within several years and develop resistance to platinum-based therapy (1). This state, termed platinum-resistant ovarian cancer (PROC), is defined as progression within 6 months of the last platinum-based regimen (2, 3). Despite advances in ovarian cancer treatment over the past decade, PROC remains a lethal disease with limited therapeutic options (2).

The current standard treatment for PROC is single-agent non-platinum chemotherapy—the most common of which are pegylated liposomal doxorubicin (PLD), paclitaxel, gemcitabine, and topotecan—with or without bevacizumab (2). As monotherapies, the clinical benefit of non-platinum single agents is modest in pretreated patients, with low objective response rates (ORR, 6%–13%), median progression-free survival (mPFS) of <6 months, and overall survival (OS) of <1 year (4–6). However, adding bevacizumab markedly increases clinical benefit (ORR, 31%; mPFS, 6.7 months; median OS [mOS], 16.6 months), particularly when combined with weekly paclitaxel (7, 8). Among tumors with *BRCA* mutations or homologous recombination deficiency (HRD), poly[adenosine diphosphate-ribose] polymerase inhibitors (PARPi) have changed the front-line treatment landscape. The role of PARPi in PROC is limited. For tumors that are PARPi naïve and *BRCA* mutated, the response rate in a single-arm study approached 30% (9). For tumors that are *BRCA* wild-type, PARPi are not considered a treatment option, as the response rate is <5% (2, 9). Most recently, mirvetuximab soravtansine, an antibody-drug conjugate (ADC) targeting folate receptor alpha (FRα), was approved by the US Food and Drug Administration (FDA) for patients with PROC and high FRα expression (10).

Bevacizumab, a monoclonal antibody that inhibits vascular endothelial growth factor (VEGF), received approval in the US and Europe in 2014 for use in combination with chemotherapy in PROC based on the results of the phase 3, randomized AURELIA trial (7, 11, 12). In AURELIA, treatment with bevacizumab combined with PLD, paclitaxel, or topotecan resulted in a longer mPFS compared to chemotherapy alone (6.7 vs 3.4 months [hazard ratio (HR), 0.48; 95% CI, 0.38–0.60; *P*<.001]) (7). Furthermore, the ORR of chemotherapy plus bevacizumab was also higher than that of chemotherapy alone (30.9% vs 12.6% [95% CI, 9.6–27.0; *P*<.001]) (7). In a correspondence published after the primary AURELIA manuscript, the paclitaxel plus bevacizumab cohort exhibited the greatest benefit, with an ORR of 53.3% (8). Interestingly, the paclitaxel monotherapy cohort performed notably well, with an ORR of 30.2% (8). Importantly, the AURELIA study population was limited to patients with ≤2 prior anticancer regimens, excluded tumors that progressed on platinum-based therapy, and included few patients with prior bevacizumab exposure (7). Single-agent bevacizumab first showed efficacy in the platinum-resistant, later-line treatment setting (13).

Bevacizumab is approved for first-line treatment of stage III/IV ovarian cancer in platinum-sensitive ovarian cancer (PSOC) and in PROC (<2 prior lines of therapy), impacting the relevance of the AURELIA data in contemporary patient cohorts (11). The AURELIA patient population may no longer accurately represent the PROC population, making response comparisons between historical and contemporary cohorts challenging.

When PROC progresses after treatment with bevacizumab-containing regimens, single-agent non-platinum chemotherapy is usually implemented (2). The efficacy of single-agent non-platinum chemotherapy with (n=52) or without (n=51) bevacizumab in Japanese patients with PROC that recurred after a bevacizumab-containing chemotherapy regimen was investigated in the open-label, randomized, phase 2 JGOG3023 trial (14). Although patients receiving chemotherapy plus bevacizumab had a numerically greater mOS than those receiving chemotherapy alone (15.3 vs 11.3 months [HR, 0.67; 95% CI, 0.38–1.17; *P*=.1556]) and a higher ORR (25.0% vs 13.7% [*P*=.0599]), the results were not significant, likely due to a small sample size (14).

Recent phase 3 trials investigating novel regimens (CORAIL, JAVELIN Ovarian 200, NINJA, FORWARD I) failed to meet their primary efficacy endpoints, highlighting the challenges of treating PROC (**Table 1**) (6, 15–17). These trials enrolled pretreated patients (≥1 prior line of therapy) with prior bevacizumab exposure ranging from 26%–49% (6, 15–17). Response rates in control groups that received standard-of-care, single-agent chemotherapy ranged from 4%–13%, with a mPFS between 3.5–4.4 months, and a mOS between 11–13 months (**Table 1**) (6, 15–17).

**Table 1:** Recent phase 3 trials in patients with platinum-resistant recurrent ovarian cancer comparing single-agent chemotherapy to an experimental agent^a^.

Trial	Prior Lines of Therapy	Endpoints	Study Population	Agent	ORR, %	mPFS, months	mOS, months
**CORAIL (6)**	1–3 prior lines of chemotherapy	Primary: PFS (IRC) Secondary: PFS (IA), OS, ORR, DOR (IRC)	90% serous; 53% (primary), 48% (secondary) platinum-resistant; 46% prior BEV; *BRCA* mutation status assessed	PLD or topotecan (n=221)	12.7% (95% CI, 8.6–17.8)	3.6 (95% CI, 2.7–3.8)	10.9 (95% CI, 9.3–12.5)
			82% serous; 50% (primary), 50% (secondary) platinum-resistant; 40% prior BEV; *BRCA* mutation status assessed	Lurbinectedin (n=221)	14.5% (95% CI, 10.1–19.5)	3.5 (95% CI, 2.1–3.7)	11.4 (95% CI, 9.0–14.2)
**JAVELIN Ovarian 200 (15)**	1–3 prior lines of chemotherapy	Primary: PFS (BICR), OS Secondary: ORR, DOR, disease control (BICR and IA)	71% high-grade serous; 75% platinum-resistant; 28% prior BEV; PD-L1 and CD8 expression assessed	PLD (n=190)	4.0% (95% CI, 2.0–8.0)	3.5 (95% CI, 2.1–4.0)	13.1 (95% CI, 11.8–15.5)
			65% high-grade serous; 75% platinum-resistant; 26% prior BEV; PD-L1 and CD8 expression assessed	Avelumab + PLD (n=188)	13.0% (95% CI, 9.0–19.0)	3.7 (95% CI, 3.3–5.1)	15.7 (95% CI, 12.7–18.7)
			72% high-grade serous; 75% platinum-resistant; 34% prior BEV; PD-L1 and CD8 expression assessed	Avelumab (n=188)	4.0% (95% CI, 2.0–8.0)	1.9 (95% CI, 1.8–1.9)	11.8 (95% CI, 8.9–14.1)
**NINJA (16)**	≥1 prior regimen after platinum-resistance diagnosis	Primary: OS Secondary: PFS, BOR, ORR, DOR, TTR (IA)	60% serous; 100% platinum-resistant; PD-L1 expression and *BRCA* mutation status assessed	Gemcitabine or PLD (n=159)	13.2% (95% CI, 7.6–20.8)	3.8 (95% CI, 3.6–4.2)	12.1 (95% CI, 9.3–15.3)
			64% serous; 100% platinum-resistant; PD-L1 expression and *BRCA* mutation status assessed	Nivolumab (n=157)	7.6% (95% CI, 3.5–13.9)	2.0 (95% CI, 1.9–2.2)	10.1 (95% CI, 8.3–14.1)
**FORWARD I (17)**	1–3 prior lines of anticancer therapy	Primary: PFS (BICR) Secondary: ORR (BICR), OS	97% high-grade serous; 100% platinum-resistant; 47% prior BEV; FRα expression ≥75% (10x staining) required; *BRCA* mutation status assessed	IC chemotherapy (n=118)	12.0%	4.4	11.8
			99% high-grade serous; 100% platinum-resistant; 49% prior BEV; FRα expression ≥75% (10x staining) required; *BRCA* mutation status assessed	Mirvetuximab soravtansine (n=248)	22.0%	4.1	NR
**MIRASOL (18, 19)**	1−3 prior lines of anticancer therapy	Primary: PFS (IA) Secondary: ORR (IA), OS, PROs	100% high-grade serous; 100% platinum-resistant; 63% prior BEV; FRα expression ≥75% (PS2+ staining) required; *BRCA* mutation status assessed	IC chemotherapy (n=226)	15.9% (95% CI, 11.4–21.4)	4.0 (95% CI, 2.9–4.5)	12.8 (95% CI, 10.9–14.4)
			100% high-grade serous; 100% platinum-resistant; 61% prior BEV; FRα expression ≥75% (PS2+ staining) required; *BRCA* mutation status assessed	Mirvetuximab soravtansine (n=227)	42.3% (95% CI, 35.8–49.0)	5.6 (95% CI, 4.3–6.0)	16.5 (95% CI, 14.5–24.6)

a This table is not exhaustive.

BEV, bevacizumab; BICR, blinded independent central review; BOR, best overall response; *BRCA*, BReast CAncer gene; CD8, cluster of differentiation 8; DOR, duration of response; FRα, folate receptor alpha; IA, investigator assessed; IC, investigator’s choice; IRC, Independent Review Committee; mOS, median overall survival; mPFS, median progression-free survival; NR, not reached; ORR, objective response rate; OS, overall survival; PD-L1, programmed cell death ligand 1; PFS, progression-free survival; PLD, pegylated liposomal doxorubicin; PROs, patient-reported outcomes; PS2+, positive staining 2+; TTR, time to tumor response.

## 2 Drug development in platinum-resistant ovarian cancer

**Figure 1:**
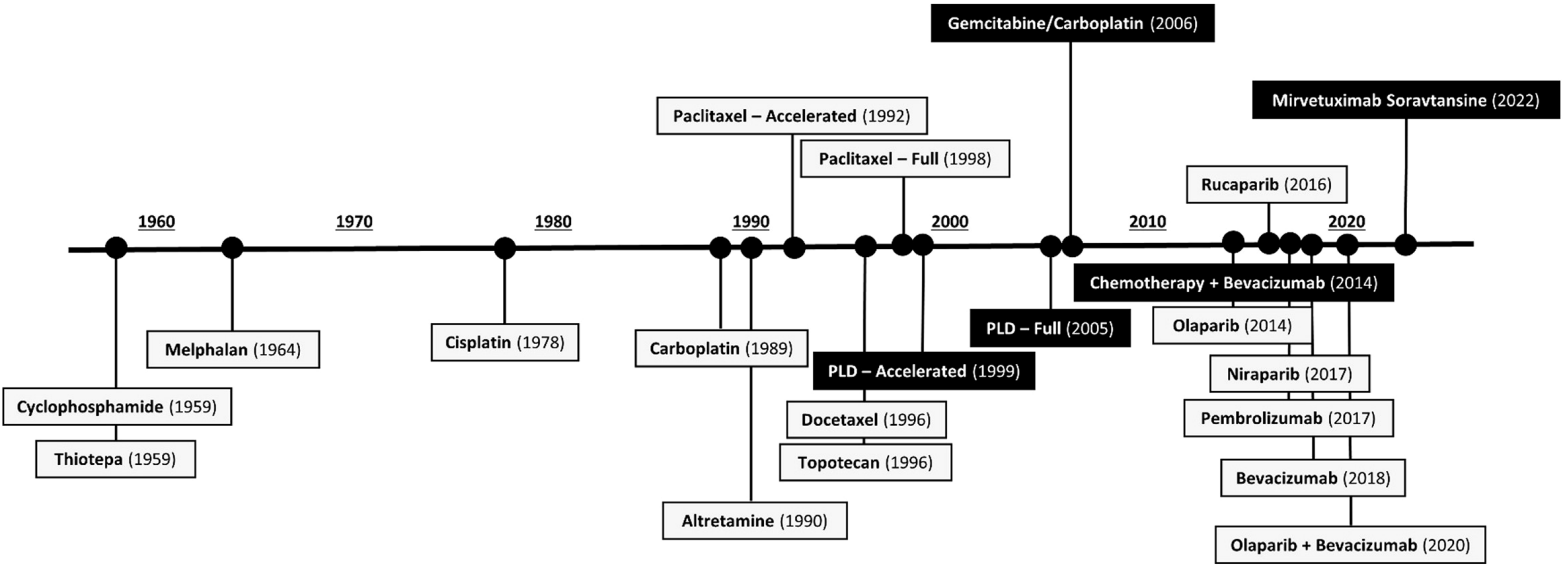
FDA-Approved Agents in Ovarian Cancer by Initial Approval Date (10, 20, 21). **Figure 1** shows all drugs approved (and currently approved, as of November 2022) for the treatment of ovarian cancer. Agents in black boxes are indicated for the treatment of platinum-resistant ovarian cancer. FDA, US Food and Drug Administration; PLD, pegylated liposomal doxorubicin.

Since the breakthroughs of paclitaxel, topotecan, and PLD in the 1990s, ovarian cancer treatments stagnated until 2014. Bevacizumab and PARPi transformed the front-line treatment of high-grade epithelial ovarian cancer, resulting in multiple new FDA approvals granted between 2014–2020 (**Figure 1**) (12). This shifted the ovarian cancer treatment paradigm to include combination and maintenance regimens after initial diagnosis and surgical resection. Conversely, 1 PROC treatment has been approved since the approval of bevacizumab plus chemotherapy in 2014 (10, 12).

Limited efficacy signals in PROC clinical trials underscore the challenge of drug development for these patients, and the implications of prior bevacizumab or PARPi exposure on response are unclear. Trials evaluating combination treatment with immunotherapy and chemotherapy in PROC also yielded disappointing results. Lurbinectedin, a cytotoxic agent that inhibits oncogenic transcription, was evaluated vs chemotherapy (PLD or topotecan) in the open-label, phase 3 CORAIL trial in patients with PROC, including those with prior bevacizumab exposure (lurbinectedin arm, 40%; PLD/topotecan arm, 46%) (6). CORAIL failed to meet the primary endpoint of significant improvement in PFS between lurbinectedin and PLD/topotecan (mPFS, 3.5 vs 3.6 months [*P*=.6059]) (6). JAVELIN Ovarian 200, an open-label, phase 3 trial evaluating avelumab (anti–programmed cell death-ligand 1 [PD-L1]) monoclonal antibody) combined with PLD vs PLD alone, was not superior in mPFS (3.7 vs 3.5 months [HR, 0.78; repeated 93.1% CI, 0.59–1.24; one-sided *P*=.030]) or mOS (15.7 vs 13.1 months [HR, 0.89; repeated 88.85% CI, 0.74–1.24; one-sided *P*=.21]) (15). Similarly, trials evaluating single-agent immune checkpoint inhibitors have reported either modest or statistically insignificant benefits, with response rates below 13% (15, 16, 22). NINJA, an open-label, phase 3 trial of the anti–programmed cell death 1 protein (PD-1) monoclonal antibody nivolumab vs chemotherapy, failed to demonstrate any statistical difference in OS (16). Indeed, mOS in the nivolumab arm was shorter compared with chemotherapy (10.1 vs 12.1 months [HR, 1.0; 95% CI, 0.8–1.3; *P*=.808]) (16). Trials evaluating monotherapies (eg, PARPi, immunotherapies) in PROC have also yielded disappointing results (**Table 2**) (9, 22, 23, 25, 27, 28). In the open-label, phase 2 KEYNOTE-100 trial in patients with advanced recurrent ovarian cancer, the anti-PD-L1 monoclonal antibody pembrolizumab showed a modest ORR of 7.4% in cohort A (1–3 prior regimens; platinum-free interval [PFI] or treatment-free interval [TFI] 3–12 months) and 9.9% in cohort B (4–6 prior regimens; PFI or TFI ≥3 months) (22). However, a prespecified analysis of KEYNOTE-100 found that higher PD-L1 expression (combined positive score ≥10) correlated with higher ORR, regardless of clinical features such as number of prior treatment lines and degree of platinum sensitivity (22).

**Table 2:** Select monotherapy clinical trials with results in heavily pretreated patients with platinum-resistant ovarian cancer.

Trial/ Phase	Treatment (N)	Study Population	Endpoints	Outcome by Platinum Sensitivity		
				Overall	Resistant	Sensitive
PARPi						
Study 42 subgroup Phase 2 (23)	Olaparib (N=137)	• Histology data not reported • Germline *BRCA1/2*-mutated advanced ovarian cancer • ≥3 prior lines of chemotherapy • Platinum-resistant (59%) • Platinum-refractory (10%) • Platinum-sensitive (28%)	ORR	34.0% (95% CI, 26.0–42.0)	30.0% (95% CI, 20.0–41.0)	46.0% (95% CI, 30.0–63.0)
			DOR	mDOR=7.9 months (95% CI, 5.6–9.6)	mDOR=8.0 months (95% CI, 4.8–14.8)	mDOR=8.2 months (95% CI, 5.6–13.5)
			PFS	mPFS=6.7 months (95% CI, 5.5–7.6)	mPFS=5.5 months (95% CI, 4.2–6.7)	mPFS=9.4 months (95% CI, 6.7–11.4)
Study 10 and ARIEL2 Phase 2 (24)	Rucaparib (N=106)	• 92% serous • *BRCA1/2*-mutated high-grade ovarian carcinoma • ≥2 prior lines of chemotherapy • ≥3 prior lines of chemotherapy • Platinum-resistant (19%) • Platinum-sensitive (75%)	ORR (IA)	53.8% (95% CI, 43.8–63.5)	25.0% (95% CI, 8.7–49.1)	65.8% (95% CI, 54.3–76.1)
			DOR (IA)	mDOR=9.2 months (95% CI, 6.6–11.6)	NA	NA
ARIEL4 Phase 3 (25, 26)	Rucaparib (N=233)	• 89% serous • *BRCA1/2*-mutated relapsed ovarian cancer • ≥2 prior lines of chemotherapy • Platinum-resistant (51%) • Platinum-sensitive (49%)	PFS (IA)	mPFS=7.4 months (95% CI, 6.7–7.9)	mPFS=6.4 months (95% CI, 5.5–7.4)	mPFS=12.9 months (95% CI, 9.2–14.8)
			OS	mOS=19.4 months	NA	NA
QUADRA Phase 2 (9)	Niraparib (N=63)	• Metastatic, relapsed, high-grade serous epithelial ovarian cancer (100%) • HRD-positive • ≥3 prior lines of chemotherapy • Platinum-resistant (33%) • Platinum-refractory (35%)	ORR (IA)	15.0%	10.0%	26.0%
Immunotherapy (anti-PD-1)						
UMIN000005714 Phase 2 (27)	Nivolumab (N=20)	• 75% serous • Platinum-resistant ovarian cancer • ≥2 prior lines of chemotherapy • ≥4 prior lines of chemotherapy • PD-L1 expression assessed	ORR (ICR)	15.0% (95% CI, 3.2–37.9)	15.0% (95% CI, 3.2–37.9)	NA
			PFS	3.5 months (95% CI, 1.7–3.9)	3.5 months (95% CI, 1.7–3.9)	NA
			OS	20.0 months (95% CI, 7.0–NR)	20 months (95% CI, 7.0–NR)	NA
KEYNOTE-100 Phase 2 (22)	Pembrolizumab Cohort A: N=285 Cohort B: N=91	• 75% high-grade serous • Advanced recurrent ovarian cancer • Cohort A: 1–3 prior lines with PFI or TFI 3–12 months • Cohort B: 4–6 prior lines with PFI or TFI ≥3 months • Platinum-resistant recurrent (38%) • PD-L1 expression assessed	ORR	Cohort A: 7.4% (95% CI, 4.6–11.0) Cohort B: 9.9% (95% CI, 4.6–17.9)	Both cohorts: 7.8% (95% CI, 4.0–13.5)	NA
			PFS	Cohort A: mPFS=2.1 months (95% CI, 2.1–2.2) Cohort B: mPFS=2.1 months (95% CI, 2.1–2.6)	NA	NA
			OS	Cohort A: mOS=NR (95% CI, 16.8–NR) Cohort B: mOS=17.6 months (95% CI, 13.3–NR)	NA	NA

*BRCA*, BReast CAncer gene; DOR, duration of response; HRD, homologous recombination deficiency; IA, investigator assessed; ICR, independent central review; mDOR, median duration of response; mOS, median overall survival; mPFS, median progression-free survival; NA, not available; NR, not reached; ORR, objective response rate; OS, overall survival; PARPi, poly (adenosine diphosphate-ribose) polymerase inhibitors; PD-1, programmed cell death 1 protein; PD-L1, programmed cell death ligand 1; PFI, platinum-free interval; PFS, progression-free survival; TFI, treatment-free interval.

## 3 Opportunities and challenges

Numerous unsuccessful clinical trials in PROC underscore the need to identify effective treatments. Novel combinations using existing therapies may improve outcomes. Recent phase 2 and 3 clinical trials of combination therapies are summarized in **Table 3**.

**Table 3:** Recent clinical trials using combination therapy approaches in pretreated patients with platinum-resistant ovarian cancer.

Trial/ Phase	Treatment (N)	Study Population	Efficacy
Chemotherapy plus antiangiogenic agent			
NCT00913835 (29) Phase 2	Olaratumab + PLD (n=62) vs PLD (n=61)	Platinum-refractory or platinum-resistant^a^ advanced ovarian cancer, histology data not reported 1–3 prior platinum-containing regimens PDGFRα expression assessed	Primary: PFS Olaratumab + PLD, mPFS=4.2 months PLD, mPFS=4.0 months HR=1.04 (95% CI, 0.70–1.56); *P*=.837 Secondary: OS Olaratumab + PLD, mOS=16.6 months PLD, mOS=16.2 months HR=1.10 (95% CI, 0.71–1.71); *P*=.678
TRIAS (30) Phase 2	Topotecan + sorafenib (n=83) vs topotecan + placebo (n=89)	Platinum-resistant ovarian cancer, 79% serous (overall population) ≤2 prior lines of therapy for recurrent disease	Primary: PFS (IA) Topotecan + sorafenib, mPFS=6.7 months Topotecan + placebo, mPFS=4.4 months HR=0.60 (95% CI, 0.43–0.83); *P*=.0018
AEROC (31) Phase 2	Apatinib + oral etoposide (N=35)	Platinum-refractory or platinum-resistant ovarian cancer, 77% high-grade serous	Primary: ORR 54.2% (95% CI, 36.6–71.2); 0 CR, 19 PR Secondary: PFS and DOR mPFS=8.1 months (95% CI, 2.8–13.4) mDOR=7.4 months (95% CI, 2.3–12.0)
GOG Protocol 260 study (32) Phase 2	IV elesclomol + weekly paclitaxel (N=56)	Platinum-resistant, recurrent, or persistent epithelial ovarian cancer, 79% serous 1 prior platinum-containing regimen	Primary: ORR 19.6% (90% CI, 11.4–30.4); 1 CR, 10 PR Secondary: PFS and OS mPFS=3.6 months mOS=13.3 months
Chemotherapy plus agents targeting DNA damage repair			
NCT01164995 (33) Phase 2	Carboplatin + AZD1775 (adavosertib) (N=23)	Platinum-refractory or platinum-resistant^b^ ovarian cancer, *TP53*-mutated, 70% serous All received prior first-line platinum + paclitaxel-based therapy Mutations in *WEE1-*related genes assessed	Primary: ORR 43.0% (95% CI, 22.0–66.0); 1 CR, 8 PR Secondary: PFS and OS mPFS=5.3 months (95% CI, 2.3–9.0) mOS=12.6 months (95% CI, 4.9–19.7)
NCT02151292 (34) Phase 2	Gemcitabine + adavosertib (n=65) vs gemcitabine + placebo (n=34)	Platinum-refractory or platinum-resistant recurrent ovarian cancer, 100% high-grade serous (main cohort) Unlimited prior lines *BRCA* and *TP53* mutation status assessed	Primary: PFS Gemcitabine + adavosertib, mPFS=4.6 months (95% CI, 3.6–6.4) Gemcitabine + placebo, mPFS=3.0 months (95% CI, 1.8–3.8) HR=0.55 (95% CI, 0.35–0.90); *P*=.015
NCT02595892 (35) Phase 2	Gemcitabine + berzosertib (n=34) vs gemcitabine (n=36)	Platinum-resistant ovarian cancer, 100% high-grade serous (overall population) Unlimited prior lines of cytotoxic therapy in platinum-sensitive setting; up to 1 prior line in platinum-resistant setting *BRCA* mutation status assessed	Primary: PFS Gemcitabine + berzosertib, mPFS=22.9 weeks (90% CI, 17.9–72.0) Gemcitabine, mPFS=14.7 weeks (90% CI, 9.7–36.7) HR=0.57 (90% CI, 0.33–0.98); *P*=.044 Secondary: ORR Gemcitabine + berzosertib, 3% Gemcitabine, 11%
Anti-PD-1/PD-L1 combination therapy			
NRG GY003 (36) Phase 2	Nivolumab (n=49) vs nivolumab + ipilimumab (n=51)	Recurrent or persistent ovarian cancer (platinum-resistant and platinum-sensitive included), 84% high-grade serous (overall population) 1–3 prior lines PD-L1 expression assessed	Primary: ORR Nivolumab, 12.2%; 3 CR, 3 PR Nivolumab + ipilimumab, 31.4%; 3 CR, 13 PR Secondary: PFS and OS Nivolumab, mPFS=2.0 months Nivolumab + ipilimumab, mPFS=3.9 months HR=0.53 (95% CI, 0.34–0.82); *P*=.004 Nivolumab, mOS=21.8 months Nivolumab + ipilimumab, mOS=28.1 months HR=0.79 (95% CI, 0.44–1.42); *P*=.43
TOPACIO/KEYNOTE-162 (37) Phase 1/2	Pembrolizumab + niraparib (N=62)	Platinum-resistant recurrent ovarian cancer, histology data not reported ≤5 prior lines PD-L1 expression, *BRCA* mutation status, and HRD status assessed	Primary: ORR (IA) Total population: 18.0% (90% CI, 11.0–29.0); 3 CR, 8 PR Platinum-resistant: 21.0% (90% CI, 9.0–37.0) ≥3 prior lines: 11.0% (90% CI, 4.0–24.0) Prior bevacizumab exposure: 19.0% (90% CI, 9.0–33.0) Secondary: PFS Total population, mPFS=3.4 months (95% CI, 2.1–5.1)
PARPi plus antiangiogenic agent			
CONCERTO (38) Phase 2b	Cediranib + olaparib (N=60)	Platinum-resistant recurrent ovarian cancer without germline *BRCA1/2* mutation, 90% high-grade serous ≥3 prior lines Somatic *BRCA* mutation status and mutation of HRR-related genes assessed	Primary: ORR (ICR) 15.3% (95% CI, 7.2–27.0); 1 CR, 1 PR Secondary: PFS and OS mPFS=5.1 months (95% CI, 3.5–5.5) mOS=13.2 months (95% CI, 9.4–16.4)
BAROCCO (39) Phase 2	Cediranib + olaparib (continuous) or cediranib + olaparib (intermittent) vs paclitaxel (n=41, each arm)	Platinum-resistant high-grade epithelial ovarian cancer, 84% serous (overall population) ≥1 prior line *BRCA* mutation status assessed	Primary: PFS Continuous, mPFS=5.6 months Intermittent, mPFS=3.8 months Paclitaxel, mPFS=3.1 months Continuous vs paclitaxel, HR=0.76 (90% CI, 0.50–1.14); *P*=.265 Intermittent vs paclitaxel, HR=1.03 (90% CI, 0.68–1.55); *P*=.904 Secondary: ORR Continuous, 15.0% Intermittent, 11.0% Paclitaxel, 38.0%
Antibody-drug conjugate plus antiangiogenic agent			
FORWARD II (40) Phase 1b	Mirvetuximab soravtansine + bevacizumab (N=94)	FRα-positive platinum-resistant epithelial ovarian, fallopian tube, or primary peritoneal cancer ≥1 prior line	Primary: ORR 44% (95% CI, 33–54); 5 CR, 36 PR Secondary: DOR and PFS mDOR=9.7 months (95% CI, 6.9–14.1) mPFS=8.2 months (95% CI, 6.8–10.0)

a Platinum-resistance was defined in this trial as disease recurrence within 12 months of platinum-based chemotherapy.

b Platinum-resistance was defined in this trial as disease recurrence within 3 months of first-line platinum-based chemotherapy.

*BRCA*, BReast CAncer gene; CR, complete response; DOR, duration of response; FRα, folate receptor alpha; GOG, Gynecologic Oncology Group; HR, hazard ratio; HRD, homologous recombination deficiency; HRR, homologous recombination repair; IA, investigator assessed; ICR, independent central review; IV, intravenous; mDOR, median duration of response; mOS, median overall survival; mPFS, median progression-free survival; ORR, objective response rate; OS, overall survival; PARPi, poly (adenosine diphosphate-ribose) polymerase inhibitor; PD-1, programmed cell death 1 protein; PD-L1, programmed cell death ligand 1; PDGFRα, platelet derived growth factor alpha; PFS, progression-free survival; PLD, pegylated liposomal doxorubicin; PR, partial response; *TP53*, tumor protein 53.

Antiangiogenic strategies have been pivotal in treating ovarian cancer, and combination therapies show promising results (3). TRIAS, a phase 2 clinical trial that evaluated a sequential combination of sorafenib—a non-selective oral multi-kinase inhibitor of VEGF—with topotecan, was the first to significantly improve OS in patients with PROC or platinum-refractory ovarian cancer (ie, cancer that progressed during platinum therapy) (30). PFS also significantly improved (30). In the open-label, phase 2b CONCERTO trial, the combination of olaparib and cediranib—an antiangiogenic targeting VEGF—demonstrated modest efficacy (ORR, 15.3%; mPFS, 5.1 months; mOS, 13.2 months) in heavily treated patients (all ≥3 prior lines of therapy) with non-germline-*BRCA1/2*-mutated PROC (38). However, this combination failed to demonstrate superior PFS vs standard-of-care paclitaxel in the open-label, phase 2 BAROCCO trial in a less heavily treated (60%, ≥3 prior lines of therapy) PROC population, of whom 12% had a *BRCA* mutation (39).

Immunotherapeutic approaches may hold promise in ovarian cancer; however, combination strategies using immune checkpoint inhibitors have yielded modest results to date (3). In the open-label, phase 2 NRG GY003 trial, patients with recurrent or persistent ovarian cancer (PFI <12 months and PROC) who received a 4-dose induction of combination nivolumab/ipilimumab followed by nivolumab exhibited a significantly higher objective response than those receiving nivolumab alone (31.4% vs 12.2% [odds ratio, 3.28; 85% CI, 1.54–infinity; *P*=.034]); however, mPFS remained low with and without nivolumab/ipilimumab induction (3.9 vs 2.0 months [HR, 0.53; 95% CI, 0.34–0.82; *P*=.0041]) (36). Despite the short induction, combination nivolumab/ipilimumab treatment yielded more grade ≥3 treatment-related adverse events (TRAEs), including increased pancreatic/liver enzymes, anemia, and colitis or diarrhea (36). The open-label, phase 1/2 TOPACIO/KEYNOTE-162 trial enrolled patients with recurrent PROC (n=30; 48%) or those deemed ineligible for platinum-based therapy (37). The immune checkpoint inhibitor/PARPi combination of pembrolizumab/niraparib demonstrated efficacy across all study populations (ORR overall, 18%; ORR PROC, 21%), irrespective of *BRCA* or HRD status, prior bevacizumab, or tumor mutational burden (37). Several chemotherapy/immunotherapy combinations are currently under investigation, in addition to trials combining anti-PD-1/PD-L1, antiangiogenic therapy, and DNA-damaging agents (3, 41). The phase 2 OPAL trial investigating dostarlimab/bevacizumab/niraparib combination has shown clinical activity in patients with PROC, most of whom had *BRCA* wild-type or HRD test negative tumors (42). The phase 2 MOONSTONE/GOG-3032 trial of niraparib plus dostarlimab did not reach the threshold for second-stage accrual at interim analysis due to a low ORR (29.3%) (43). The phase 2 CAPRI trial showed that olaparib plus ceralasertib yielded no objective responses (ie, complete or partial), but showed improved survival outcomes in a subgroup of patients with *BRCA* mutations (44).

Lastly, the phase 1b FORWARD II trial investigated the combination of the ADC mirvetuximab soravtansine with bevacizumab and found encouraging efficacy (ORR, 44% [95% CI, 33–54]), comparable to standard of care (40).

Of note, baseline clinical characteristics such as number of prior lines of chemotherapy, prior treatment regimens (eg, bevacizumab), histologic subtype, and mutational status are important to consider when interpreting efficacy outcomes in PROC. Therefore, much of this information has been described in **Tables 1**-**3**, which report outcomes in relevant clinical trials in PROC.

## 4 New approaches to drug development

Addressing the unmet need in PROC requires thoughtful clinical trial designs and novel effective agents. Recent cancer drug approvals emphasize incorporating molecular phenotypes into clinical trial designs to better define patient and tumor characteristics that may benefit from the study drug. The era of precision medicine has witnessed tumor-agnostic approvals, based on expression of a common biomarker rather than simply the tumor type (defined by primary site of origin) (45). In 2017, the FDA approved pembrolizumab for unresectable/metastatic solid tumors progressing after prior treatment in adult and pediatric patients based on microsatellite instability-high status or mismatch repair deficiency (dMMR), irrespective of tumor type (46). In 2020, pembrolizumab received approval to treat adult and pediatric patients with unresectable/metastatic tumor mutational burden-high solid tumors (excluding central nervous system cancers) and progression after prior therapy (46). Similarly, larotrectinib and entrectinib, 2 selective inhibitors of tropomyosin receptor kinases, received approval for treating unresectable/metastatic solid tumors with a neurotrophic tyrosine receptor kinase gene fusion in adult and pediatric patients, regardless of tumor origin (47, 48). Additional recent tumor-agnostic approvals include dabrafenib plus trametinib (based on *BRAF V600E* or *V600K* mutations) and dostarlimab (based on dMMR) (49–51).

Unfortunately, high-grade serous ovarian cancers are not commonly associated with specific driver mutations, but rather by chromosomal instability and copy number alterations (52). Considering histology in this cancer type, while recognizing that some patients (eg, those with high FRα expression) may benefit from targeted treatment, is necessary (45). Tumor-agnostic indications may be most appropriate for drugs with high response rates and for rare tumors (45). As our understanding of ovarian cancer evolves, we must include biomarkers, companion diagnostics, and molecular profiling in clinical trial designs.

Further, umbrella and basket protocol designs can compare different novel regimens or incorporate different patient populations within 1 trial. Notably, in the investigator-initiated, phase 2 AMBITION trial in PROC, patients with HRD test positive tumors were randomized to combination olaparib/cediranib or olaparib/durvalumab, while those with HRD test negative tumors were randomized by PD-L1 expression to durvalumab/chemotherapy, durvalumab/tremelimumab at 75 mg/chemotherapy, or durvalumab/tremelimumab at 300 mg/chemotherapy (53). Additionally, the open-label, phase 2 BOUQUET trial is an ongoing biomarker-driven trial evaluating multiple biomarker-based therapies in patients with persistent or recurrent rare epithelial ovarian tumors, including but not limited to low-grade serous ovarian carcinoma, clear cell carcinoma, mucinous carcinoma, and carcinosarcoma (54). Data from this trial may inform biomarker-driven patient stratification and treatment selection.

Finally, clinical trials in the PROC setting may have limited generalizability to real-world patient populations due to eligibility criteria that restrict enrollment to moderately pretreated individuals with potentially fewer comorbidities. Additionally, some trials lack an appropriately diverse patient population, limiting our ability to understand clinical benefit across certain racial and ethnic groups. There are active initiatives to address these shortcomings by both ensuring diversity in enrollment while simultaneously mandating more practical inclusion and exclusion criteria in PROC trials.

## 5 Accelerated approvals, benefits, and pitfalls

The FDA’s accelerated approval program (and analogous non-US programs) can expedite approvals of drugs that address unmet needs for serious or life-threatening conditions (55). Accelerated approval can be based on a surrogate or intermediate clinical endpoint “reasonably likely to predict clinical benefit,” providing earlier evaluation of clinical benefit based on well-controlled phase 2 studies (55). Although OS is the most objective benchmark for demonstrating clinical benefit in oncology clinical trials, it requires a larger sample size and longer follow-up compared with more rapidly assessed endpoints (eg, time to tumor progression and PFS) (56). Since ORR can be assessed using a single-arm trial, it is the most common surrogate endpoint for accelerated approvals (57). Duration of response (DOR) is also occasionally used to support ORR (57). The gold standard for endpoints in PROC randomized trials remains OS, but when OS is confounded by long post-progression survival (>18 months) and crossover (common in trial participants), PFS is the preferred endpoint. Open-label studies where PFS is a key endpoint should utilize placebo controls and blinded independent central review to objectify clinical activity.

Several treatments for ovarian cancer have benefitted from accelerated approval (58). PLD’s accelerated approval occurred in 1999 based on 3 phase 2 studies, with confirmation from a randomized phase 3 trial (58, 59). However, some accelerated approvals in oncology do not demonstrate clinical benefit in confirmatory studies (57). Notably, bevacizumab received accelerated approval for metastatic breast cancer in 2008 based on improved PFS in an open-label, phase 3 clinical trial (60, 61). Subsequent placebo-controlled, double-blind confirmatory trials failed to confirm that the magnitude of the effect on PFS constituted a clinical benefit and showed more TRAEs compared to chemotherapy, which led to a revoked approval for this indication (62).

In ovarian cancer, the voluntary withdrawals of rucaparib, olaparib, and niraparib for recurrent, late-line treatment demonstrate the vulnerabilities of accelerated approval (63–65). These withdrawals stemmed from non-hypothesis-tested subset analyses, which suggested a detrimental effect on OS with PARPi exposure when compared to chemotherapy.

Although post-approval studies typically take years to complete (median [range], 3.4 years [0.5–12.6]), as of November 2022, only 21 indications (for 16 different drugs) with accelerated approvals have been withdrawn, while 88 were verified in confirmatory studies (57, 66, 67). While accelerated approvals usually endure, choosing appropriate surrogate endpoints and designing proper single-arm trials that can predict meaningful treatment effects remain challenging.

## 6 Moving away from “platinum-resistant ovarian cancer”

Over the past decade, the definition of “platinum resistant” has shifted from utilizing only the historical definition of disease recurrence <6 months after the completion of last platinum-based chemotherapy (2, 68). In clinical trials and practice, PFI-based classification has been accepted for predicting chemotherapy response, disease prognosis, and patient selection and stratification (68). However, platinum response is neither binary, nor accurately represented by an arbitrary cutoff based solely on the time of diagnosis of recurrence (68, 69). Further, evidence suggests that PFI may not be optimal for predicting clinical response in all cases. Previous trials such as AURELIA have shown clinical benefit independent of PFI (7). Thus, a PFI of <6 months may not equate with resistance to platinum agents. A recent meta-analysis reported a 36% response rate to platinum-based regimens vs a 16% response rate to non-platinum-based regimens in a PROC population (70). Therefore, platinum rechallenge remains a viable option for some historically defined patients with PROC. Although the term “PROC” has evolved in clinical practice, it is still useful for regulatory approval due to previously discussed uncertainties, where platinum retreatment may not be the best option.

Several factors can affect time to relapse and platinum sensitivity. Variable timing and application of tools to detect recurrence (eg, cancer antigen-125, computed tomography scan, positron emission tomography scan) can skew the time to relapse and impact historical “platinum sensitive” vs “platinum resistant” designations (68, 69). Further, the tumor’s molecular profile can influence response. Patients with *BRCA1/2-*mutated recurrent ovarian cancer may respond to platinum-based and other chemotherapy agents that induce direct DNA damage (69, 71, 72). In ovarian cancer, the type, number, and outcome of prior therapies are important considerations when assessing potential response to further treatments (68). However, it is unclear what impact maintenance therapy with targeted and biological agents may have on subsequent treatment response (68). Other influential factors include histological subtype, prior surgical interventions, symptoms at recurrence, and the molecular profile beyond *BRCA* (eg, HRD status) (68, 69).

These factors have been incorporated in a new proposed classification system for patients with recurrent ovarian cancer and in the Gynecologic Cancer InterGroup (GCIG) consensus recommendations for recurrent ovarian cancer clinical trials (68, 69). The GCIG recommends TFI replaces PFI, with specific reporting of the TFI from last platinum dose (TFIp) and the specific method used to diagnose recurrence (68). Additionally, the GCIG recommends recording the TFI from last non-platinum therapy (TFInp) and last biological agent (TFIb), where applicable (68). The GCIG also notes that biomarkers will likely play a more predictive role than TFIp regarding treatment response (68).

Historically, developing new PROC regimens without reliable and established biomarkers for treatment response has been challenging. The recent validation of FRα as a predictive biomarker for the ADC mirvetuximab soravtansine heralds a new strategy for drug development where tumor surface antigens may be identified, quantified, and targeted. For trials targeting immune mechanisms, biomarkers such as tumor-infiltrating lymphocytes and tumor mutational burden (a tumor’s total number of somatic coding mutations) may guide which patients may respond to immunotherapy (3); however, these biomarkers have not been validated.

Including biomarkers in future clinical trials will help establish their relevance in ovarian cancer and their relationship to efficacy endpoints, including DOR. This will help identify measures to inform patient response beyond the 6-month PFI cutoff.

## 7 Future perspectives

Developing effective therapies for patients with PROC is complex and necessitates consideration of tumor and patient heterogeneity. Ongoing and recently completed clinical trials feature novel agents, which utilize combination treatments and targeted, biomarker-based therapies (**Table 4**).

**Table 4:** Ongoing and recently completed phase 2/3 trials in platinum-resistant ovarian cancer.

Therapy 1	Therapy 2		Therapies Under Investigation			
			NCT Number	Phase	Interventions	Est. Primary Completion Date
**Chemotherapy**	**Antiangiogenic agents**	Bevacizumab	NCT05310344	Phase 2, single group, open label	Albumin-bound paclitaxel + bevacizumab	March 2023
			NCT02312245	Phase 2, randomized, parallel, open label	Avatar-directed chemotherapy (gemcitabine) or avatar-directed chemotherapy (topotecan/PLD/paclitaxel) + bevacizumab	July 2023
			NCT04670978	Phase 2, single group, open label	Albumin-bound paclitaxel + bevacizumab biosimilar	December 2023
			NCT04753216	Phase 2, single group, open label	Irinotecan liposome + bevacizumab	July 2023
			NCT03632798 (ACSCO)	Phase 3, randomized, parallel	ChemoID (drug response assay) cancer stem cell assay-guided chemotherapy + bevacizumab vs standard-of-care, IC chemotherapy + bevacizumab	July 2022
		Ofranergene obadenovec (ofra-vec; VB-111)	NCT03398655	Phase 3, randomized, parallel	Ofranergene obadenovec (ofra-vec; VB-111 [adenoviral-based antiangiogenic]) + paclitaxel vs placebo + paclitaxel	December 2022
		Chiauranib	NCT04921527	Phase 3, randomized, parallel	Chiauranib (targets against VEGFR, Aurora B, and CSF-1R) + paclitaxel vs placebo + paclitaxel	July 2023
		Apatinib	NCT04348032 (APPROVE)	Phase 2, randomized, parallel, open label	Apatinib (VEGFR-2 inhibitor) + PLD vs PLD	January 2021 (actual)
		BD0801	NCT04908787	Phase 3, randomized, parallel	BD0801 (VEGFR inhibitor) + chemotherapy (paclitaxel/topotecan/PLD) vs placebo + chemotherapy	December 2023
**Chemotherapy**	**Immunotherapy agents**	Pembrolizumab	NCT02440425	Phase 2, single group, open label	Paclitaxel + pembrolizumab	April 2020 (actual)
			NCT02901899	Phase 2, single group, open label	Guadecitabine (decitabine prodrug) + pembrolizumab	April 2020 (actual)
			NCT02865811	Phase 2, single group, open label	Pembrolizumab + PLD	August 2020 (actual)
		Durvalumab	NCT03699449 (AMBITION)	Phase 2, randomized, parallel	Durvalumab + chemotherapy (1 arm)	September 2022
**Chemotherapy**	**PARPi**	Niraparib	NCT04217798	Phase 2, single group, open label	Niraparib + oral etoposide	March 2022
**Chemotherapy**	**Agents targeting DNA repair**	Berzosertib	NCT02595892	Phase 2, randomized, open label	M6620 (berzosertib; ATR inhibitor) + gemcitabine vs gemcitabine	June 2020 (actual)
		Adavosertib	NCT02101775	Phase 2, randomized, parallel	Gemcitabine +/- adavosertib (WEE1 inhibitor)	February 2022 (actual)
**Chemotherapy**	**Agents targeting AKT/ERK**	Afuresertib (ONC201)	NCT04374630	Phase 2, randomized, parallel	Afuresertib (AKT inhibitor) + paclitaxel vs paclitaxel	July 2022
			NCT04055649	Phase 2, single group, open label	AKT/ERK inhibitor ONC201 + paclitaxel	April 2023
**Chemotherapy**	**Other agents**	Batiraxcept (AVB-S6-500)	NCT04729608 (AXLerate-OC)	Phase 3, randomized, parallel	Batiraxcept (AVB-S6-500 [AXL inhibitor]) + paclitaxel vs paclitaxel + placebo	July 2023
		Relacorilant	NCT03776812	Phase 2, randomized, parallel	Relacorilant (selective glucocorticoid receptor modulator) + nab-paclitaxel vs nab-paclitaxel	March 2023
		Intraoperative hyperthermic intraperitoneal chemotherapy (HIPEC)	NCT05316181	Phase 3, randomized, open label	HIPEC + doxorubicin + mitomycin vs IC chemotherapy	December 2024
		Decitabine	NCT03467178	Phase 2, randomized, parallel, open label	Decitabine + carboplatin vs chemotherapy	December 2021
**Immunotherapy**	**Antiangiogenic agents**	Bevacizumab	NCT05116189 (MK-3475-B96/KEYNOTE-B96/ENGOT-ov65)	Phase 3, randomized, parallel	Pembrolizumab + paclitaxel ± bevacizumab vs placebo + paclitaxel ± bevacizumab (docetaxel may be used if patient is paclitaxel intolerant)	June 2025
		Anlotinib	NCT05145218	Phase 3, open label, parallel	Anlotinib (VEGFR inhibitor) + TQB2450 (anti-PD-1) vs paclitaxel	October 2024
		Apatinib	NCT04068974	Phase 2, single group	Camrelizumab (anti-PD-1) + apatinib (VEGFR-2 inhibitor)	June 2021
**Immunotherapy**	**Immunotherapy**	Durvalumab + tremelimumab	NCT03026062	Phase 2, randomized, parallel	Durvalumab + tremelimumab (sequential) vs durvalumab + tremelimumab (combination)	December 2023
		Nemvaleukin + pembrolizumab	NCT05092360	Phase 3, randomized, parallel	Nemvaleukin (engineered IL-2) + pembrolizumab vs pembrolizumab vs nemvaleukin	December 2025
		Etigilimab + nivolumab	NCT05026606	Phase 2, single group, open label	Etigilimab (anti-TIGIT) + nivolumab	May 2023
		Pembrolizumab + P53MVA	NCT03113487	Phase 2, single group, open label	Pembrolizumab + P53MVA (modified vaccinia virus Ankara vaccine expressing p53)	December 2023
		Batiraxcept (AVB-S6-500) + durvalumab	NCT04019288	Phase 1/2, randomized, parallel, open label	Batiraxcept (AVB-S6-500 [AXL inhibitor]) + durvalumab	September 2022
**PARPi**	**Antiangiogenic agents**	Cediranib	NCT02502266	Phase 2/3, randomized, parallel	Cediranib (VEGFR inhibitor) + olaparib vs cediranib vs olaparib	June 2023
			NCT03117933 (OCTOVA)	Phase 2, randomized, parallel	Cediranib + olaparib	March 2021 (actual)
		Anlotinib	NCT05130515 (CC-ANNIE)	Phase 2, single group	Niraparib + anlotinib (VEGFR-2 inhibitor)	June 2023
			NCT04376073 (ANNIE)	Phase 2, single group	Niraparib + anlotinib (VEGFR-2 inhibitor)	December 2021
		Bevacizumab	NCT05170594	Phase 2, open label	Bevacizumab + fluzoparib (PARPi) vs bevacizumab + chemotherapy vs fluzoparib	June 2024
			NCT04556071	Phase 2, single group, open label	Niraparib + bevacizumab	March 2022
**PARPi**	**Other**	Azenosertib (ZN-c3)	NCT05198804	Phase 1/2, single group, open label	Azenosertib (ZN-c3 [WEE1 inhibitor]) + niraparib	November 2023
		Cediranib or durvalumab	NCT03699449 (AMBITION)	Phase 2, randomized, parallel	Olaparib + cediranib vs durvalumab + olaparib vs durvalumab + chemotherapy	September 2022
		Alpelisib	NCT04729387	Phase 3, randomized, parallel	Alpelisib (PI3K inhibitor) + olaparib vs paclitaxel or PLD	June 2023
		Copanlisib	NCT05295589	Phase 2, randomized, parallel, open label	Olaparib + copanlisib (PI3K inhibitor) vs chemotherapy (PLD/paclitaxel/topotecan)	July 2024
		Arsenic trioxide	NCT04518501	Phase 1/2, sequential, open label	Fuzuloparib (PARPi) + arsenic trioxide	January 2024
**Antibody-drug conjugates**	**Other**	Durvalumab + BA3011	NCT04918186	Phase 2, open label	Durvalumab + BA3011 (AXL-targeted ADC) vs durvalumab + BA3021 (ROR2-targeted ADC)	June 2024
		Upifitamab rilsodotin	NCT03319628	Phase 1/2, parallel	Upifitamab rilsodotin (NaPi2b-targeted ADC)	April 2023
		Anetumab ravtansine + bevacizumab	NCT03587311	Phase 2, randomized, open label	Anetumab ravtansine (mesothelin-targeted ADC) + bevacizumab	October 2022
		Mirvetuximab soravtansine	NCT04296890	Phase 3, single group, open label	Mirvetuximab soravtansine (FRα-targeted ADC)	November 2021 (actual)
		Mirvetuximab soravtansine	NCT04209855	Phase 3, randomized, parallel, open label	Mirvetuximab soravtansine (FRα-targeted ADC) vs chemotherapy	December 2022
**Nano-drug conjugates**	**Antiangiogenic agent**	EP0057	NCT04669002	Phase 2a/b, randomized in 2b	EP0057 (camptothecin nanoparticle-drug conjugate) + olaparib vs chemotherapy	November 2022
**Bispecific antibody**		Navicixizumab	NCT05043402	Phase 3, randomized, open label	Navicixizumab (bispecific mAb targeting VEGFR and delta-like ligand 4)	November 2023
**Other**	**Other**		NCT04720807	Phase 2, single group, open label	Letrozole (aromatase inhibitor) + anlotinib (VEGFR inhibitor)	August 2022
			NCT05043402	Phase 3, randomized, open label	Navicixizumab (bispecific mAb targeting VEGFR and delta-like ligand 4) + paclitaxel vs paclitaxel	November 2023
			NCT04851834	Phase 1/2, non-randomized, open label	NTX-301 (hypomethylating agent) vs NTX-301 + platinum-based chemotherapy vs NTX-301 + temozolomide	August 2023
			NCT03949283	Phase 3, randomized, parallel	ChemoID (drug response assay) cancer stem cell assay-guided chemotherapy vs chemotherapy	June 2024
			NCT05272462	Phase 2, single group, open label	Minoxidil (anti-hypertensive agent)	December 2023
			NCT02364713	Phase 2, randomized, parallel, open label	Bevacizumab + chemotherapy vs MVNIS (oncolytic measles virus encoding thyroidal sodium iodide symporter)	March 2027
**Triplet combinations**			NCT04361370 (OPEB-01)	Phase 2, single group	Olaparib + pembrolizumab + bevacizumab	May 2026
			NCT03699449 (AMBITION)	Phase 2, randomized, parallel	Olaparib + cediranib vs durvalumab + olaparib vs durvalumab + chemotherapy vs durvalumab + tremelimumab + chemotherapy	September 2022
			NCT03363867 (BEACON)	Phase 2, single group, open label	Atezolizumab + bevacizumab + cobimetinib (MEK inhibitor)	July 2022
			NCT04739800	Phase 2, randomized, parallel, open label	Durvalumab + olaparib + cediranib vs olaparib + cediranib or cediranib + durvalumab vs standard-of-care chemotherapy (paclitaxel/PLD/topotecan)	December 2023
			NCT03206047	Phase 1/2, randomized, parallel, open label	Atezolizumab + guadecitabine + DEC-205/NY-ESO-1 fusion protein CDX-1401 vs atezolizumab + guadecitabine vs atezolizumab	March 2023
			NCT02839707	Phase 2/3, randomized, parallel, open label	PLD + atezolizumab and/or bevacizumab	June 2023
			NCT02659384	Phase 2, randomized, parallel	Bevacizumab vs bevacizumab + atezolizumab vs bevacizumab + atezolizumab + acetylsalicylic acid	February 2021
			NCT02923739	Phase 2, randomized, parallel, open label	Paclitaxel + bevacizumab ± emactuzumab (anti-CSF-1R)	May 2025
			NCT04781088	Phase 2, single group, open label	Lenvatinib (VEGFR inhibitor) + paclitaxel + pembrolizumab	December 2024

ADC, antibody-drug conjugate; CSF-1R, colony stimulating factor-1 receptor; FRα, folate receptor alpha; IC, investigator’s choice; IL-2, interleukin 2; mAb, monoclonal antibody; MEK, mitogen-activated protein kinase; NaPi2b, sodium-dependent phosphate transporter; PARPi, poly(adenosine diphosphate-ribose) polymerase inhibitor; PD-1, programmed cell death 1 protein; PLD, pegylated liposomal doxorubicin; VEGFR, vascular endothelial growth receptor.

Since ADCs have succeeded in treating other cancers, the use of this novel approach of targeting cytotoxic payloads to tumor cells is also being evaluated in PROC (73).

To date, the only ADC that has been evaluated for PROC in a pivotal trial is mirvetuximab soravtansine, an ADC that targets FRα, which is minimally expressed on normal tissues but overexpressed in >80% of epithelial ovarian tumors (73–75). In the open-label, phase 3 FORWARD I trial, mirvetuximab soravtansine did not meet its primary endpoint of PFS, but both ORR and OS favored mirvetuximab soravtansine over chemotherapy (ORR, 24% vs 10% [*P*=.014]; OS, 17.3 vs 12.0 months [HR, 0.71; 95% CI, 0.49–1.02; [*P*=.063]) in a subgroup of patients with high FRα expression, although these endpoints were not statistically significant (17). Importantly, mirvetuximab soravtansine displayed improved tolerability compared to chemotherapy; grade ≥3 TRAEs (25.1% vs 44.0%), dose reductions (19.8% vs 30.3%), and discontinuations (4.5% vs 8.3%) occurred more frequently in the chemotherapy group (17). In the pivotal, single-arm SORAYA trial of patients with FRα-high PROC, mirvetuximab soravtansine demonstrated an ORR of 32.4% (median DOR [mDOR], 6.9 months), with clinical benefit maintained across prespecified subgroups, including patients with 3 prior therapy lines (ORR, 30.2%; mDOR, 7.4 months) and with prior PARPi exposure (ORR, 38.0%; mDOR, 5.7 months) (76). The confirmatory, randomized, phase 3 MIRASOL trial evaluated the efficacy and safety profile of single-agent mirvetuximab soravtansine vs single-agent chemotherapy (77). Compared with chemotherapy, mirvetuximab soravtansine showed statistically significant improvements in investigator-assessed PFS (mPFS, 5.6 vs 4.0 months [HR, 0.65; 95% CI, 0.52–0.81; [*P*<.0001]), investigator-assessed ORR (42.3% vs 15.9% [odds ratio, 3.81; 95% CI, 2.44–5.94; [*P*<.0001]), and OS (mOS, 16.5 vs 12.8 months [HR, 0.67; 95% CI, 0.50–0.89; [*P*=.0046]) (18, 19). No new safety signals occurred with mirvetuximab soravtansine and improved tolerability with mirvetuximab soravtansine vs chemotherapy was demonstrated by comparatively fewer grade ≥3 treatment-emergent adverse events ([TEAEs] 42% vs 54%), serious adverse events (24% vs 33%), and TEAEs leading to discontinuation (9% vs 16%) (18, 19).

Additional ADCs targeting other proteins are being evaluated in earlier-stage clinical trials in PROC, including mesothelin (anetumab ravtansine), sodium-dependent phosphate transporter (NaPi2b; upifitamab rilsodotin), dipeptidase 3/DPEP3 (tamrintamab pamozirine), mucin 16/MUC16 (sofituzumab vedotin), tissue factor (tisotumab vedotin, recently approved for recurrent/metastatic cervical cancer), and Trop-2 (SKB264, datopotamab deruxtecan, sacituzumab govitecan) (73, 78–80).

Given the heterogeneity of PROC, combination therapies may improve clinical outcomes. Chemotherapy is being explored in combination with antiangiogenics, immune checkpoint inhibitors, PARPi, agents targeting DNA damage repair, agents targeting AXL and AKT/ERK, and a glucocorticoid receptor modulator (**Table 4**). Non-chemotherapeutic immunotherapy regimens being investigated include existing and novel immune checkpoint inhibitors combined with antiangiogenics, an engineered IL-2, an anti-TIGIT monoclonal antibody, and a p53 vaccine. PARPi are being studied in non-chemotherapeutic combinations with antiangiogenics and inhibitors of PI3K and WEE1. Triplet combinations utilizing various agents, including PARPi, anti-PD-1/PD-L1, and antiangiogenics, are underway. Other ongoing and recently completed phase 2 and 3 trials are summarized in **Table 4**.

These novel PROC treatments may improve response rates and prolong PFS for patients with few options. However, improving the treatment paradigm will require overcoming several obstacles. Disease heterogeneity is a major challenge that may have influenced prior trial failure (41). Identifying a well-defined homogeneous population will be critical to ensure impactful trial outcomes. Increased testing for biomarkers such as tumor mutational burden and dMMR, particularly in mucinous, clear cell, and endometrioid histologies, is encouraged, as is participation in appropriate clinical trials (81). Another challenge in PROC is the frailty of patients—due to age, comorbidities, and/or toxicity from multiple rounds of prior therapy—who are frequently recruited for trials and often have rapidly progressive disease (68). In 1 study, 30%–50% of patients discontinued due to progressive disease before receiving sufficient doses of therapy, and therefore had no chance of clinical benefit (15). Focusing on more specific populations may improve outcomes by excluding patients unlikely to be positively impacted.

## 8 Conclusion

Addressing the unmet need for effective therapies in heavily pretreated PROC is an ongoing challenge. As research elucidates the molecular mechanisms of ovarian cancer progression, new insights will provide guidance on developing novel targeted therapies.

## Author contributions

RE: Writing – review and editing, conceptualization. KM: Writing – review and editing, conceptualization. BM: Writing – review and editing, conceptualization. TH: Writing – review and editing, conceptualization. CA: Writing – review and editing, conceptualization. DO: Writing – review and editing, conceptualization. RC: Writing – review and editing, conceptualization.
